# Improving the Generalizability of Deep Learning for T2-Lesion Segmentation of Gliomas in the Post-Treatment Setting

**DOI:** 10.3390/bioengineering11050497

**Published:** 2024-05-16

**Authors:** Jacob Ellison, Francesco Caliva, Pablo Damasceno, Tracy L. Luks, Marisa LaFontaine, Julia Cluceru, Anil Kemisetti, Yan Li, Annette M. Molinaro, Valentina Pedoia, Javier E. Villanueva-Meyer, Janine M. Lupo

**Affiliations:** 1 Department of Radiology and Biomedical Imaging, UCSF, San Francisco, CA 94143, USA; jacob.ellison@ucsf.edu (J.E.); francesco.caliva@ucsf.edu (F.C.); pablo.damasceno@ucsf.edu (P.D.); tracy.luks@ucsf.edu (T.L.L.); marisa.lafontaine@ucsf.edu (M.L.); julia.cluceru@ucsf.edu (J.C.); anil.kemisetti@ucsf.edu (A.K.); yan.li@ucsf.edu (Y.L.); valentina.pedoia@ucsf.edu (V.P.); javier.villanueva-meyer@ucsf.edu (J.E.V.-M.); 2 Center for Intelligent Imaging, UCSF, San Francisco, CA 94143, USA; 3 UCSF/UC Berkeley Graduate Program in Bioengineering, San Francisco, CA 94143, USA; 4 Department of Neurological Surgery, UCSF, San Francisco, CA 94143, USA; annette.molinaro@ucsf.edu

**Keywords:** glioma, post-treatment, segmentation, deep learning

## Abstract

Although fully automated volumetric approaches for monitoring brain tumor response have many advantages, most available deep learning models are optimized for highly curated, multi-contrast MRI from newly diagnosed gliomas, which are not representative of post-treatment cases in the clinic. Improving segmentation for treated patients is critical to accurately tracking changes in response to therapy. We investigated mixing data from newly diagnosed (*n* = 208) and treated (*n* = 221) gliomas in training, applying transfer learning (TL) from pre- to post-treatment imaging domains, and incorporating spatial regularization for T2-lesion segmentation using only T2 FLAIR images as input to improve generalization post-treatment. These approaches were evaluated on 24 patients suspected of progression who had received prior treatment. Including 26% of treated patients in training improved performance by 13.9%, and including more treated and untreated patients resulted in minimal changes. Fine-tuning with treated glioma improved sensitivity compared to data mixing by 2.5% (*p* < 0.05), and spatial regularization further improved performance when used with TL by 95th HD, Dice, and sensitivity (6.8%, 0.8%, 2.2%; *p* < 0.05). While training with ≥60 treated patients yielded the majority of performance gain, TL and spatial regularization further improved T2-lesion segmentation to treated gliomas using a single MR contrast and minimal processing, demonstrating clinical utility in response assessment.

## 1. Introduction

Gliomas are the most common form of primary central nervous system (CNS) tumors in adults [1,2], comprising 81% of all malignant CNS tumors in the US with 21,440 average annual cases from 2016 to 2020 [3]. The prognosis of this cancer varies, with five-year median survival rates ranging from 5% to 73% [4,5,6] and expected survival projections reaching as low as 6 months in the most aggressive cases of glioblastoma (GBM) [4]. To assess response to treatment, spatial measurements from anatomical MRI, including T1-weighted images pre- and post-injection of a gadolinium-based contrast agent, T2-weighted images, and T2 Fluid Attenuated Inversion Recovery (FLAIR) images, are typically used in response assessment according to the Response Assessment in Nero-Oncology (RANO) criteria [7]. These criteria are still based on the 2D product of maximum bidimensional diameters of contrast-enhancing tumor and qualitative evaluation of T2 FLAIR hyperintensity [7] and suffer from inter- and intra-rater variability associated with their manual determination, which are improved using fully automated volumetric assessments. Volumetric approaches are also more correlated to patient outcomes and are more sensitive to subtle longitudinal changes [8,9,10,11].

Although progression is typically defined on T1-weighted contrast-enhanced imaging [12] for IDH-wildtype and higher grade IDH-mutant gliomas, the importance of tracking changes in the volume of T2 FLAIR hyperintensity is apparent in terms of both response assessment in non-enhancing tumors [7] and its ability to predict overall survival in post-operative glioma more accurately [13] because T2 FLAIR hyperintensity represents, in part, tumor infiltration into normal brain tissue [14,15]. As new therapies emerge and subsequent imaging biomarkers are established, quantifying volumetric changes in both T1 post-contrast and T2 FLAIR tumor volume for clinical evaluation and treatment planning is essential.

Deep neural networks have provided rapid, highly accurate segmentations for newly diagnosed, often treatment-naïve gliomas [8,9,16,17,18,19,20,21,22,23,24,25,26,27,28,29,30,31,32,33,34]. Currently, the most prevalent and high-performing methods in the computer vision literature for automated tumor segmentation use a variety of encoder–decoder-based architectures in an end-to-end approach to generate lesion masks directly from MR image inputs [30,32,35]. These methods are primarily inspired by the pioneering developments of convolutional neural networks (CNNs) capable of 3D segmentation in works like U-net [19] and V-net [20], with more recent advances employing techniques such as multi-task learning [22,23,24], generative modeling for augmenting training data or adversarial approaches [26,36,37,38,39,40,41], hybrid machine learning approaches [27,42], domain adaptation and transfer learning [29,43,44,45,46,47,48,49,50,51,52,53], task-specific loss modification [18,25,27,31,34,54], diffusion models [41,55,56,57], and attention mechanisms like transformer modules [58,59,60], as well as federated learning approaches [34,61,62,63] to improve performance.

Despite the promising segmentation accuracy and time savings when employing deep learning models in untreated patients, the same level of accuracy has not been reported after treatment, with previous work showing decreased accuracy in post-treatment anatomy [37], and a greater frequency of failure to generate lesion annotations after treatment [8]. This is partly because many of the prevailing neural networks for brain tumor lesion segmentation require four anatomical imaging contrasts to segment even just the T2 lesion, and they have been optimized for performance in the ongoing Multimodal Brain Tumor Segmentation (BraTS) challenge dataset for Adult Glioma [16,28], which currently consists of radiologist-annotated images from newly diagnosed glioma patient scans before surgery or treatment. Leading performances of T2-lesion segmentation models have reported Dice scores of the whole tumor above 0.9 and 95th Hausdorf Distances below 3 mm [64]. T2 FLAIR hyperintense lesions from newly diagnosed gliomas, however, tend to have better boundary signal delineation from healthy tissue and lack the subtle variations in contrast observed compared to surrounding normal-appearing brain that results from adjuvant treatment or disease progression [65]. The utility of deep learning models used in monitoring longitudinal tumor progression and treatment response [10,11] is directly dependent on the accuracy of these models to perform well on treated gliomas. Although a few more recent studies have achieved equivalent performance in segmenting treated gliomas [66,67,68,69], they still either require multiple (4) image contrasts as input to segment multiple tumor compartments simultaneously, necessitate multiple image preprocessing steps (i.e., co-registration/skull stripping), use very few post-operative patients for training and testing, neglect edema and infiltration seen on T2-weighted images, or report low Dice scores (<0.65).

To overcome these challenges, this study took a multi-pronged approach to evaluate strategies for developing a more practical tool for segmentation of the T2 hyperintense lesion at time points relevant to clinical practice by (1) using only T2 FLAIR images as input; (2) employing a robust but easily implemented variational autoencoder network (VAE) model architecture that won the BraTS challenge in 2018 [21]; (3) training on a diverse dataset of pre-treatment patients and post-treatment patients at the time of suspected progression; (4) applying transfer learning from pre- to post-treatment imaging domains; and (5) employing spatial distance-based loss function weightings to specifically improve segmentation performance near low contrast lesion boundaries where prior models have struggled.

## 2. Materials and Methods

### 2.1. Patient Data

3D T2 FLAIR images (TE: 114 ms–127 ms, TR: 5850 ms–6400 ms, ETL:148–200, 1 × 1 × 1.5 mm resolution; additional details in Appendix A) acquired on 3T GE MR 750 scanners from 429 patients with glioma (208 newly diagnosed; 221 post-treatment with no overlap) were retrospectively used to train and evaluate modified versions of NVIDIA’s 2018 BraTS challenge winning VAE [21] to predict manually annotated T2-hyperintense lesions from a single input image. Extra care was taken to ensure that the labels of our testing dataset of 24 post-treatment patients were as accurate as possible by using multiple expert readers for review. Segmentation masks were generated manually by TLL, reviewed by JE, and then further confirmed or revised as needed by both JEV-M and JML. Unlike the BraTS dataset, the images were not skull-stripped before training. This was carried out to eliminate a preprocessing step that can introduce variability dependent on the brain extraction algorithm and allow for more versatile training strategies in situations where skull stripping may not be available. The imaging data were all acquired at UCSF within 48 h before a patient underwent surgical resection. All previously treated patients had undergone prior standard-of-care treatment, which included surgical resection, and various combinations of radiation and chemotherapy. As a result, these lesions also contained a mixture of recurrent tumor and treatment-induced injury, which is typical of what is observed in clinical practice. All patients provided informed consent for their images to be used in research.

### 2.2. Network Architecture and Hyperparameters

**Figure 1:**
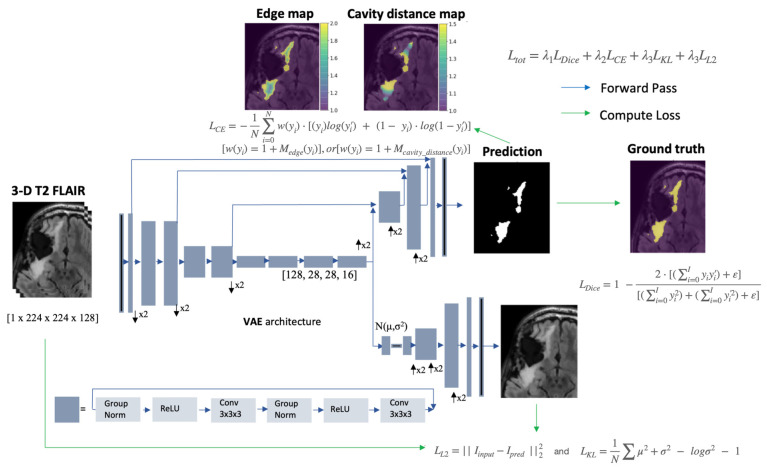
Modified VAE [21] architecture with larger crop size, single input/output channel, fewer convolutional kernels, and smaller latent space. Spatial weighting penalizations for the cross-entropy term and overall loss function are shown.

Our network was adapted from the NVIDIA GPU Cloud catalog. The original network consisted of a modified V-net with a variational branch that encodes the original input image during training and employed Kullback–Leibler (KL) divergence and L2 loss [21]. We modified the network’s configuration to use a single contrast 3D T2 FLAIR image input and generate only the T2-hyperintense lesion mask (and reconstruct a single channel only using the VAE branch) in contrast to the original configuration requiring four image contrasts as input, resulting in a three-channel lesion segmentation output. Crop size in preprocessing was increased from [160 × 192 × 128] to [224 × 224 × 128] to retain potential contextual structures relevant to segmentation and ensure the vast majority of the image remained since only one input channel was used. This led to a smaller overall encoding size of the network ([128 × 28 × 28 × 16] instead of [256 × 20 × 24 × 16]). Sixteen initial filters were used instead of 32 to fit into the available GPU capacity. Otherwise, the network architecture and hyperparameters were unchanged compared to the original model [21]. A schematic of the modified network architecture is shown in Figure 1.

### 2.3. Preprocessing and Augmentation

Random center cropping from [256 × 256 × 140] to [224 × 224 × 128], random spatial flipping, normalizing non-zero intensities, and scale shifting intensities from the original VAE model [21] were applied as pre-transforms during training. Distance maps were generated before training to reduce the computational cost of implementing the additional spatial boundary penalizations described in Appendix B. These maps were used only to compute the loss and thus only needed to be generated for training and not while using the model for inference.

### 2.4. Loss Function

The loss function was modified to penalize the network based on spatial weighting schemes utilizing these distance maps. Drawing heavily from the work carried out by Caliva et al. [70] this was accomplished by incorporating a weighted cross entropy term into the total loss function by adding it to Dice loss and the two VAE branch penalty terms, KL divergence and *L*_2_. Each term was added with weightings of λ_3_, λ_2_ = 0.1, and λ_1_ = 1.0 to form the final loss function:

(1) $$L_{tot}=\lambda_{1}L_{Dice}+\lambda_{2}L_{CE}+\lambda_{3}L_{KL}+\lambda_{3}L_{L2},$$

(2) $$L_{CE}=-\frac{1}{N}\sum_{i=0}^{N}{w(y}_{i})\cdot [{(y}_{i})log(y_{i}^{\prime })+(1-y_{i})\cdot log(1-y_{i}^{\prime })],$$

where *w*(*y_i_*) is the weighting defined by the spatial relationship of each pixel to either the boundary of the T2 lesion [$w(y_{i})=1+M_{edge}(y_{i})]$ or the post-surgical resection cavity [$w(y_{i})=1+M_{cavity\_distance}(y_{i})]$ shown in Figure 1. One is added to these terms to mitigate the problem of vanishing gradients. This operation is performed during the preprocessing of the distance maps, as shown in Appendix B. These modifications were chosen to handle the imbalance of edge pixels and bias the network towards the non-geometric and heterogenous borders of T2 lesions on FLAIR images that are distinctive of gliomas post-treatment. Code for generating the spatial regularization weightings and applying the calculation to the loss can be found at https://github.com/LupoLab-UCSF/SpatialRegularization (accessed on 13 March 2024).

### 2.5. Training

Training was first performed on 192 newly diagnosed patients using NVIDIA’s Clara-Train software (v1-v3) on two V100-32GB GPUs. The proportion of training images from treated gliomas was then increased while maintaining 80%/20% train/validation splits. Models were tested on a separate set of 24 patients with post-treatment glioma. Next, training was performed with training/validation/test splits beginning with 153/39/24 volumes from treated patients and successively adding 50 newly diagnosed patients into training/validation until 208 were included. The exact training dataset breakdown for these experiments is shown in Table 1. Models were trained for 300 epochs, and the models with the highest Dice scores in validation were selected for testing.

Transfer learning (TL) experiments were performed with a combined ratio of 25/167 post- to pre-treatment patients in training using the same hyperparameters. This ratio was systematically increased to 128/64 post- to pre-treatment patients, and another model was trained using the same dataset and hyperparameters but instead using the TL approach. The TL model was first pre-trained on the newly diagnosed data for 300 epochs and then fine-tuned for another 300 epochs with the same proportion of post-treatment patients as the combined model. The TL and combined models were trained deterministically using the same random seeds. Models with the highest Dice scores in validation were selected for testing. The training breakdown for these experiments is shown in Table 2.

To compare the effects of using spatial loss weightings, models were first trained using the 197 post-treatment patients with edge loss, cavity distance loss, and standard Dice loss, each three times using different random seeds. Evaluation metrics were averaged across each patient from models produced from all initializations. These models were compared while fine-tuning our pre-treatment model that was trained for 300 epochs with a training/validation split of 166/42 patients. Next, using the different loss functions, our pre-treatment model was fine-tuned with all 197 of the post-treatment patients. These models were compared to the TL model that was fine-tuned with the standard loss function and the model trained on the total combined dataset of pre- and post-treatment patients with the standard loss function. Training was performed over 300 epochs, and the models with the highest Dice scores in validation were selected for testing. These models were also trained three times with different random seeds, and the evaluation metrics for each patient in the test set were averaged for each model produced by the different initializations and compared. If a seed diverged before 10,000 steps for either spatially weighted loss model, a new one was chosen for all methods. Training curves are shown in Appendix C.

### 2.6. Evaluation Metrics

Models were evaluated based on mean Dice score, 95th percentile Hausdorff Distance (HD) [71], sensitivity, and specificity compared to the radiologist annotation of the post-treatment test set of 24 patients. The Dice score was calculated as $\frac{2\cdot [(\sum_{i=0}^{I}y_{i}y_{i}^{\prime })+\varepsilon ]}{\left[(\sum_{i=0}^{I}y_{i}^{2})+(\sum_{i=0}^{I}y_{i}^{\prime 2})+\varepsilon \right]}$, with top and bottom smoothing values of ε=0.001 during the evaluation of the test set to prevent penalization of segmentations that were correctly predicted as containing no lesion. These values were set to 0 and 1 × 10^−5^ in training. Since the calculation of Dice weights all voxels evenly, it may not adequately reflect segmentation accuracy at the lesion boundary, which is most important when defining longitudinal changes after treatment. As traditional HD, defined as the maximum distance between the set of nearest points between two objects [71], has been shown to provide a more reflective metric of boundary errors and shape by matching segmentation shape near the boundary, but can be oversensitive to outliers [71,72,73], we quantified the 95th percentile HD using a DeepMind implementation [74] in conjunction with Dice score, sensitivity, and specificity to enable evaluation of overall segmentation accuracy and more focused shape evaluation at the lesion boundary. Wilcoxon signed-rank tests implemented with SciPy 1.9.1 were used to test for statistical significance between methods, averaging metrics across models from experiments repeated using three seeds. Slicer4 was used to visualize segmentation mask overlays.

## 3. Results

### 3.1. Data Mixing

**Figure 2:**
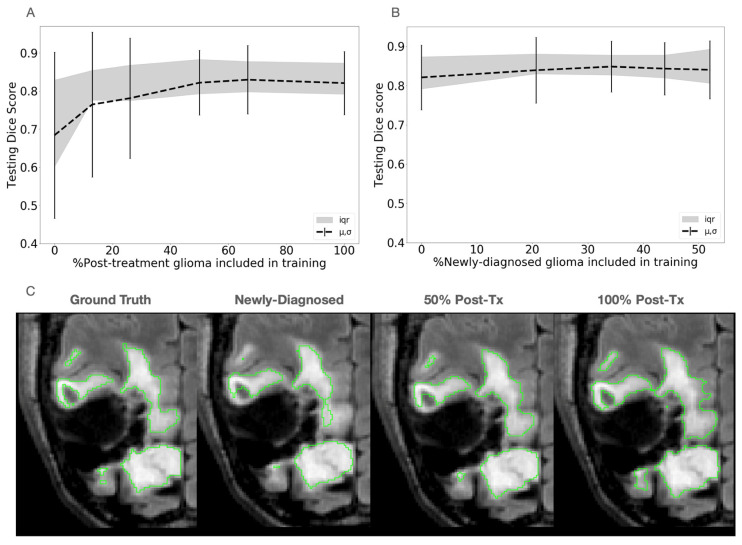
Effect of mixing pre- and post-treatment data into training. (**A**) Fixed 192 patients with an increasing ratio of post-Tx glioma (*x*-axis plots the percentage of patients with treated gliomas that were included in the training set with respect to the total number of treated and treatment-naïve patients). (**B**) Starting with 192 post-Tx gliomas and adding intervals of 50 newly diagnosed gliomas until equal proportions (*x*-axis plots the percentage of patients with newly diagnosed, treatment-naïve gliomas that were included in the training set with respect to the total number of treated and treatment-naïve patients). (**C**) Example comparison of segmentation masks (green contours) at test time with different proportions of post- and pre-treatment data in training. The post-treatment domain-specific model appears to capture areas near the edges of the lesion with lower hyperintensities on the T2 FLAIR images than the newly diagnosed or mixed domain-trained model for this case.

With no post-treatment patients included in the training, the model performed segmentation of the T2 lesion on the treated test set with an average Dice score of 0.68. After 26% of post-treatment patients (60 patients) were included in training with the total number of patients fixed, the Dice scores sharply increased by 13.9% to 0.78, and then gradually increased with greater post-treatment patient ratios until plateauing at 0.82. A similar trend was observed in the 95th HD, with an initial mean value of 26.4 mm to 12.1 mm after 26% inclusion and a steady improvement of 8.3 mm. Dice scores remained steady with a slight improvement to 0.84 when starting with 192 post-treatment patients in training and adding intervals of 50 newly diagnosed patients, with the corresponding 95th HD following a similar trend, improving from 8.3 mm to 7.1 mm. These changes in performance patterns with the inclusion of post-treatment patients into the training set can be seen in Figure 2A,B; example segmentations are shown in Figure 2C.

### 3.2. Transfer Learning

**Figure 3:**
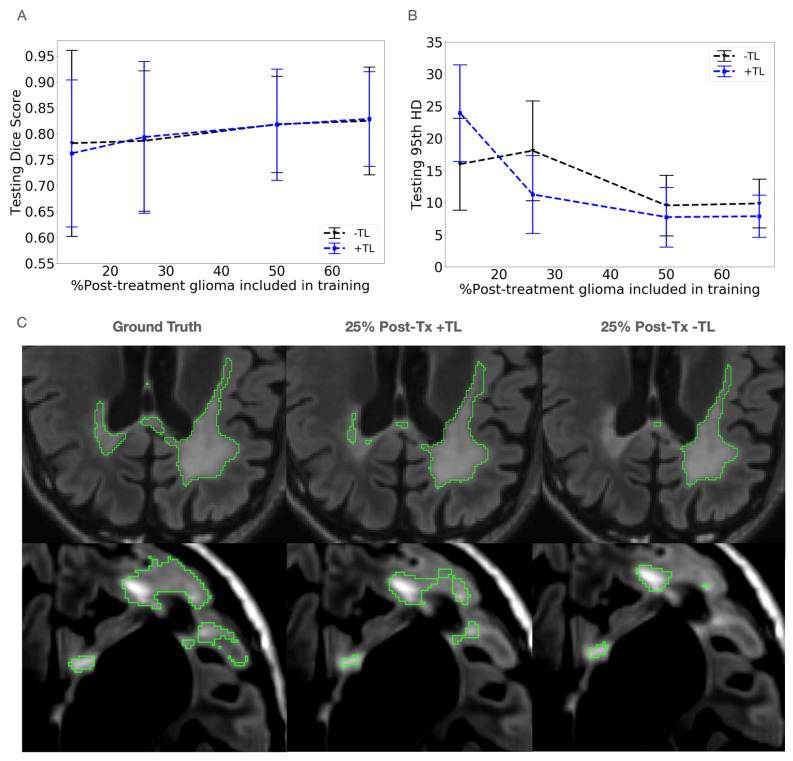
Comparison of testing Dice scores (**A**) and 95th percentile Hausdorff Distances (HD) (**B**) for a fixed number of patients (192) with varied training strategies of combined training or transfer learning (TL) at different ratios of post-treatment patients in training. There was little difference in the Dice scores but an observable improvement in the 95th HDs using the TL strategy. (**C**) Example comparisons of segmentation using TL with a ratio of 25% post- to pre-treatment data. Example comparison of segmentation masks at test time for transfer learning (TL) and no TL with a ratio of 25% post- to pre-treatment data. Fine-tuned model to post-treatment domain appears to better capture areas of the lesion near the opposite ventricle (**C** Top Row) and near edges of the lesion with lower hyperintensities on the T2 FLAIR images (**C** Bottom Row) for these cases.

Throughout the increase in the proportion of treated patients into training (13%, 26%, 50%, and 67%), the models initialized with newly diagnosed patients and fine-tuned with post-treatment patients showed little difference in Dice score compared to their combined trained counterparts, as shown in the plot in Figure 3A. The transfer learning models increased in mean Dice score from 0.78 to 0.83, while the combined trained counterpart models increased from 0.76 to 0.83. While the difference in Dice scores was minimal, the mean 95th HD was improved in the TL models (from 18.0 mm to 11.3 mm) following 26% inclusion of post-treatment patients. This trend continued through 66% inclusion of post-treatment patients (improvement of HD from 9.9 mm to 7.9 mm), with the difference in the 95th HD between the two methods decreasing as the post-treatment dataset size was increased. This trend is shown in Figure 3B, with the resulting segmentations shown in Figure 3C.

### 3.3. Loss Modification

**Figure 4:**
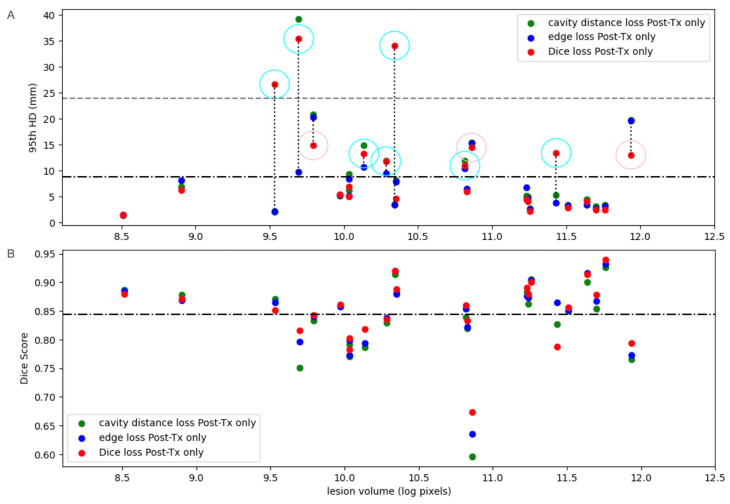
(**A**) Testing 95th percentile Hausdorff Distances (HD)s of models trained with varied loss functions on post-treatment data only and ordered by lesion size. The mean for all models is shown in dashed and dotted lines and two times the standard deviation as a dashed line. Samples for the edge loss models are connected to the same samples for Dice loss models with dotted lines. Samples above the mean 95th HD are encircled in cyan when edge loss is better and pink when Dice loss is better. Spatial weightings show a reduction in extreme errors with HD metric on post-treatment data. (**B**) Testing Dice scores of models trained with varied loss functions on post-treatment data only. Models using spatial distance-based regularization did not improve Dice scores. Both distance-weighted loss models had significantly lower Dice scores (*p* < 0.05), and the cavity distance loss had significantly higher 95th HD (*p* < 0.05).

For models trained only on 192 post-treatment patients, incorporating spatial weightings in the loss function did not improve overall performance. Both the cavity distance and edge-weighted loss models had significantly lower Dice scores (*p* = 0.004, *p* = 0.007), and the cavity distance loss had substantially higher 95th HD (*p* = 0.015). However, there was notable disagreement between the 95th HD and Dice scores for six patients in the test set, indicating a reduction in extreme errors by the models trained with spatial weightings, as shown in Figure 4. When the model was trained with Dice loss alone (plus the conventional KL and L2 terms), it resulted in very high 95th HDs when the model incorrectly segmented normal tissue located far away from the lesion, where signal gradients mimicked those of the less hyperintense signal present at the leading edge of the lesion. Training with edge loss improved 95th HD for 42% of patients, whereas training with cavity distance loss improved 95th HD for 21% of test patients compared to models trained with Dice alone.

**Figure 5:**
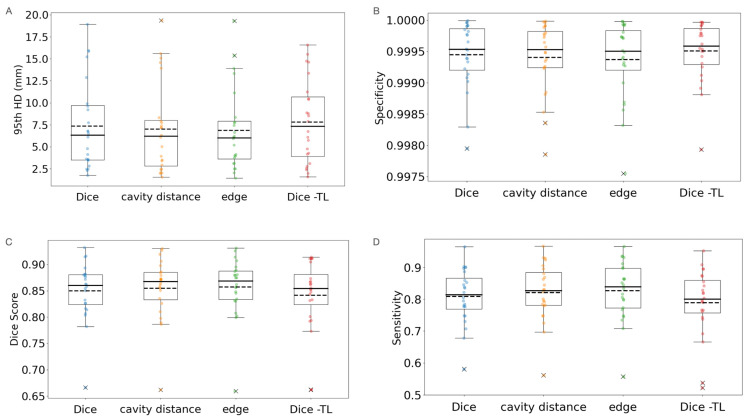
(**A**) Testing 95th percentile Hausdorff Distances (HD)s, (**B**) specificities, (**C**) Dice scores, and (**D**) sensitivities of transfer learning (TL) models initialized on pre-treatment data and then fine-tuned with varied loss functions on post- and pre-treatment data. The metrics are averaged across three seeds for each model. Means are shown in dotted lines, and medians are solid lines. Spatial weightings show improved HD, Dice, and sensitivity metrics on post-treatment data when used with TL, and TL showed improvement over combined training.

For models fine-tuned with post-treatment data using variable loss functions, a significant improvement in the 95th HD was observed both for models fine-tuned with edge loss and cavity distance loss when compared to the combined trained models using standard Dice loss (from 7.8 mm to 6.9 mm for edge loss, *p* = 0.02; and from 7.8 mm to 7.0 mm for cavity loss, *p* < 0.05) as shown in Figure 5A. Utilizing a cavity distance loss function significantly reduced 95th HD compared to fine-tuning with Dice loss alone (7.4 mm; *p* = 0.03). The Dice scores for the models trained with cavity distance (0.85) and edge loss (0.86) were also significantly higher than the combined (0.84; *p* = 0.02, *p* = 0.004) and TL models (0.85; *p* < 0.02, *p* = 0.002) trained with Dice alone (Figure 5B). The sensitivity of the models trained with cavity distance (0.82) and edge loss (0.83) was also significantly improved compared to the combined (0.79; *p* < 0.0002, *p* = 0.00005) and TL models (0.81; *p* = 0.006, *p* = 0.0005) trained with Dice alone, and the model fine-tuned with Dice alone was significantly more sensitive than the combined training with Dice (*p* = 0.02), as shown in Figure 5C. Although a significantly inverse trend was also observed for specificity, the differences between models were all less than 0.00015. A summary of all performance metrics combined is reported in Table 3.

**Figure 6:**
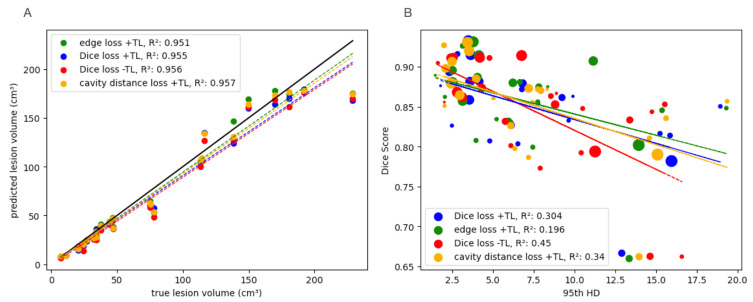
(**A**) All models fine-tuned on post-treatment anatomy or trained on a combination with a sufficiently large quantity of post-treatment patients showed significantly good agreement with the true lesion volumes, indicating utility for volumetric tracking in response to treatment. R-squared values shown are all significant (*p* < 0.05). Slopes: Dice loss: 0.9001, edge loss: 0.9431, cavity distance loss: 0.9281, Dice loss -TL: 0.8997. (**B**) Disagreement between evaluation metrics for each TL model. The size of the points indicates the size of the lesion. The best-performing model overall (edge loss with transfer learning (TL)) showed the lowest correlation between the Dice score and the 95th Hausdorff Distance (HD), indicating the importance of using both metrics to evaluate model performance. Smaller lesions appear more susceptible overall to deviations from the correlations in metrics. R-squared values shown are all significant (*p* < 0.05). Slopes; Dice loss: −5.95 × 10^−3^; edge loss: −5.30 × 10^−3^; cavity distance loss: −6.54 × 10^−3^; Dice loss -TL: −9.75 × 10^−3^.

All models trained using pre- and post-treatment patients showed significantly high correlations between predicted lesion volume and the actual lesion volume shown in Figure 6A. This indicates high consistency with manual definitions of volume critical for longitudinal tracking. For these models, there were relatively low correlations between 95th HD and Dice metrics, with the best-performing model fine-tuned on post-treatment data with edge-weighted loss, which had the lowest correlation between the two metrics, as shown in Figure 6B.

## 4. Discussion

As the vast majority of model development for brain tumor segmentation still focuses on using publicly available datasets that mainly consist of newly diagnosed or post-surgery (but still prior to adjuvant treatment) MR imaging, unsurprisingly, these models do not generalize well when applied clinically in the post-treatment setting, where their utility is needed most for monitoring response. This study sheds light on the dichotomy between typical training sets utilized and utility for clinical implementation, offers insight into effectively leveraging the widespread availability of pre-treatment data with smaller amounts of post-treatment data, and demonstrates the benefit of incorporating relatively simple but effective modifications to training strategies to tailor T2-lesion segmentation of gliomas to effectively monitor response to treatment. Overall, our model achieved a performance that was on par with results from similar deep learning-based studies of segmenting gliomas post-treatment as shown in Table 4, while using a single MR contrast and minimal processing [10,66,67,68,75,76,77,78].

From our data mixing experiments, we found that including 60 post-treatment images in training greatly improved the accuracy of the models in testing on post-treatment patients within this distribution. Less performance gain was observed from including additional post-treatment patients in training or increasing the total number of patients in training. This supports the hypothesis that there is a domain shift between post- and pre-treatment images that must be addressed when training deep learning models for application to treated patients and that training with ~30% post-treatment patients will aid in the generalization of T2-lesion segmentation models to the post-treatment setting.

Our results also support using domain-specific fine-tuning instead of training on larger, more diverse datasets for this task. Although transfer learning appeared to improve 95th HDs compared to the combined training approach at most mixing ratios, the highest performance gains were observed when the number of post-treatment patients in the training set was roughly 25%, which is similar to the findings of Ghaffari et al. [69]. Sensitivity, Dice scores, and 95th HD were also improved when fine-tuning on all available post-treatment patients after initially training on all available pre-treatment patients as opposed to the combined training approach. This supports TL as a valuable method for domain adaptation to treated patients, specifically in situations where the model can only access a few treated training examples. This insight is particularly relevant for federated learning, a strategy for which models are trained across many private datasets without moving the data away from the institution [35,61,62,63,78,79]. Our experiments support the idea that if there is a significant shift in the task imaging domain, or there is not sufficient representation of the desired domain, it may be helpful to then fine-tune a model to a more specific dataset tailored to the clinical use case, as was seen with post-treatment T2-lesion segmentation.

Although the models trained only on the post-treatment images with loss functions that include relevant spatial information did not perform as well as those trained using Dice loss alone, they did limit outliers in the 95th HD. For the 24 patients in the test set, models trained with spatial distance weighted loss functions performed substantially better in the cases where 95th HD was greater than 8 mm, as shown in Figure 4A (three times for edge loss and four times for cavity loss). The difference in performance was due to the prevention of misclassification of lesion voxels farther away from the primary lesion, where using the distance-based penalizations reduced the number of high errors by the 95th HD. This supports the idea that spatial distance loss modifications could be employed to improve regularization for T2-lesion segmentation and limit outliers, resulting in significantly improved performance when using these loss functions in the transfer learning context. This is likely due to the added regularization encouraging higher sensitivity to the domain of treated patients when fine-tuning, while preventing overfitting to the more specific post-treatment validation sets.

Our experiments illustrated an interesting discrepancy in performance metrics, highlighting the importance of careful selection and interpretation of evaluation metrics for segmentation and the limitations of the Dice coefficient as a standard metric for segmentation model evaluation. In the TL experiments, the difference in Dice scores when comparing TL or combined training models was smaller than the differences in the 95th HD, with a low correlation between the two metrics, even for the best-performing model. Similarly, for models optimized with post-treatment data alone, the Dice scores did not reflect the outliers found by the 95th HD. These discrepancies highlight the different characteristics these metrics capture when determining the performance of segmentation models. For example, when the segmentation model correctly identifies the bulk of the stable portion of a large lesion, but much smaller areas away from the lesion are misclassified as new progression or volume increases, an elevated 95th HD would capture this error. In contrast, a high Dice coefficient in this scenario would be entirely inaccurate because it would be clinically considered a failure, misclassifying a lesion as progressed when it was stable. Thus, for lesion segmentation for treated gliomas, 95th HD should have a heavy focus for evaluation in addition to Dice, as it may be a more contextually relevant metric since it focuses on the segmentation object boundary, which is the portion of the lesion of utmost importance in determining disease progression and notoriously most challenging to distinguish on T2 FLAIR images.

**Figure 7:**
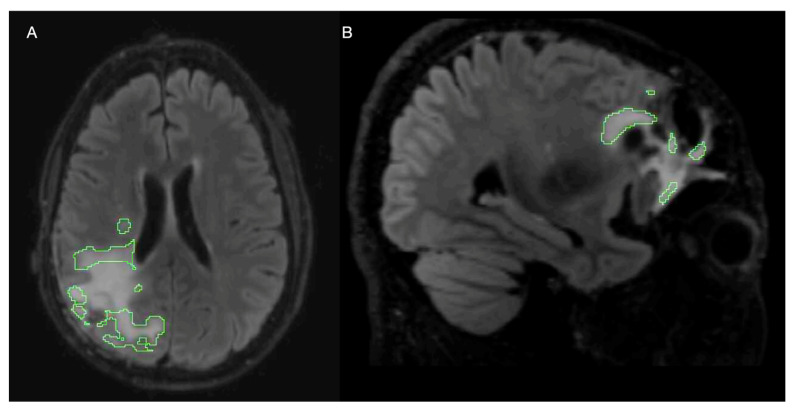
Examples of model-generated segmentation masks converging toward extreme weighting of edges from modification to the loss function: (**A**) an axial view of a sample at test time for a model trained with edge weighted loss, which was evaluated quantitatively for performance; (**B**) a sagittal view of a sample from an edge-weighted model that diverged during training and was not included in the quantitative evaluation of performance.

Several limitations arose from this study. First, the method to generate cavity regions for the spatial distance weighting scheme for the cavity distance cross-entropy loss term was highly variable. However, this may have resulted in an unintended benefit of incorporating a similar effect to label smoothing into training. As label smoothing acts as added regularization that accounts for variability in ground truth annotations, it can improve robustness to overconfidence and overfitting when applied to image classification and segmentation [80]. Although not systematically evaluated in this study, we speculate that by including imperfect distance maps compared to manually annotated cavity labels, we inserted randomness into the weighting scheme that supports additional regularization, resulting in improved performance. The same seeds were used to train each model for model comparison. When combining transfer learning with loss modification, some initializations diverged, suggesting that adapting to a new image domain and loss function with the same learning rate as the initial task could be too drastic, leading to divergence under specific random seeds. This divergence also occurred while not using transfer learning, indicating the need for a more extensive loss weighting search. A visual example from a model weighted by the edge loss that diverged during training with transfer learning and edge loss vs. one that did not diverge is shown in Figure 7. Although this pattern of the model neglecting the central portion of the lesion was also sometimes observed when using Dice loss alone, it became more severe when using spatial penalizations, and we believe it ultimately caused divergence in some instances. This behavior may also serve to demonstrate the strength of the edge-weighting approach in that it can identify challenging-to-segment, low-contrast T2-lesion creep that is characteristic of tumor progression, which occurs despite this model’s overall inability to recognize the more visually apparent portion of the lesion (which is less clinically significant). This observation warrants further exploration of approaches for the optimal combination of multiple models, the configuration of loss functions to adequately capture both these regions in one model, and a more extensive search space of hyperparameters for performance.

Although the benefits that measuring the entire lesion volume can provide in routine response assessment and prognosis are well acknowledged in the neuro-oncology community, volume calculations have still not been adopted into clinical practice because of the lengthy times they take to manually define using commercially available software and the unreliable measures often provided by automated algorithms. The proposed method would improve response assessment to therapy in neuro-oncology by providing volume measurements of the entire lesion, which is especially critical in clinical trials as well as for noticing subtle changes in size serially.

Despite the growing success of segmentation-based deep learning models for this task, there are still several barriers limiting their clinical translation due to (1) a > 25% failure rate when incorporated into clinical workflow [8], and (2) poor generalizability to images acquired after treatment. The former can be attributed to (1) the requirement of four different types of anatomic images (T2, T2-FLAIR, and T1 pre- and post-contrast); (2) lengthy preprocessing that includes alignment, reformatting a pre-determined resolution in an axial orientation, and extraction of brain tissue; and (3) the lack of a well-integrated clinical deployment system. The latter brings up the limitation that the vast majority of model development for brain tumor segmentation still focuses on using publicly available datasets, which mainly consist of newly diagnosed or post-surgery (but still prior to adjuvant treatment) MR imaging. Unsurprisingly, these models do not generalize well when applied clinically in the post-treatment setting, where their utility is most needed for monitoring response. Generalizability is commonly evaluated broadly by assessing performance across institutions, patient populations, and disease time points. In the context of post-treatment T2-lesion segmentation, our results suggest that instead of measuring success by performance across a larger or more diversified dataset, evaluating the model’s performance on a more specific dataset that better represents the clinical use case more accurately reflects true generalizability. The approach taken here aims to make the most efficient use of sparse datasets to fine-tune performance specifically for the intended use case for inference.

## 5. Conclusions

This study demonstrated the benefits of applying three different training strategies for improving the generalizability of segmenting the T2-hyperintense lesion from T2 FLAIR images to treated gliomas without requiring four aligned MR contrasts and skull stripping like most segmentation models. The best performance was achieved when pre-training with newly diagnosed data, followed by fine-tuning with post-treatment data using a model that incorporates a distance-based added penalization. These results also highlight the benefit of fine-tuning the model specifically to post-treatment patients as a training strategy for increasing generalizability and employing the 95th-percentile Hausdorff Distance as an evaluation metric for future studies that specifically segment the T2 lesion of gliomas in the post-treatment setting. Current efforts are underway to incorporate our models in real-time in the clinic for prospective validation and quantification of serial changes in tumor volumes.

## Figures and Tables

**Table 1:** Data mixing splits.

Pre-Tx Training	Pre-Tx Validation	Post-Tx Training	Post-Tx Validation	Post-Tx/Total	Pre-Tx/Total	Training Total
153	39	0	0	0	--	192
134	33	20	5	0.13	--	192
114	28	40	10	0.26	--	192
77	19	77	19	0.5	--	192
51	13	103	25	0.67	--	192
0	0	153	39	1	0	192
40	10	153	39	--	0.21	242
80	20	153	39	--	0.34	292
120	30	153	39	--	0.44	342
166	42	153	39	--	0.52	400

**Table 2:** Transfer learning and loss function training splits.

	Pre-Tx Training	PreTx Validation	Post-Tx Training	Post-Tx Validation	Post-Tx/Total	Training Total	TL Pre-Train	TL Fine-Tune
TL splits	134	33	20	5	0.13	192	167	25
	114	28	40	10	0.26	192	172	50
	77	19	77	19	0.5	192	96	96
	51	13	103	25	0.67	192	64	128
Loss splits	--	--	158	39	--	--	--	--
TL + Loss splits	166	42	158	39	0.49	405	208	197

**Table 3:** Summary of results for comparing loss functions for different training strategies with means ± 95% confidence intervals.

**Spatial Weighting Post-Treatment Only**	**Dice Loss**	**Cavity Loss**	**Edge Loss**	
Dice score	0.849 ± 0.011	0.837 ± 0.013	0.845 ± 0.012	
95th HD	10.23 ± 1.81	8.97 ± 1.60	7.23 ± 0.98	
Sensitivity	0.811 ± 0.016	0.786 ± 0.020	0.802 ± 0.019	
Specificity	0.99940 ± 0.00010	0.99948 ± 0.00012	0.99945 ± 0.00012	
**Transfer Learning (TL) + Spatial Weighting**	**TL + Dice Loss**	**TL + Cavity Loss**	**TL + Edge Loss**	**no TL + Dice Loss**
Dice score	0.850 ± 0.010	0.855 ± 0.011	0.857 ± 0.011	0.842 ± 0.013
95th HD	7.35 ± 0.96	7.00 ± 0.97	6.88 ± 0.88	7.81 ± 0.89
Sensitivity	0.809 ± 0.016	0.821 ± 0.017	0.827 ± 0.017	0.789 ± 0.02
Specificity	0.99945 ± 0.00010	0.9994 ± 0.00012	0.99937 ± 0.00012	0.9995 ± 0.00012

**Table 4:** Summary of current performance of state-of-the-art post-treatment deep learning glioma segmentation.

Study	Tumor Component	Dice Score	HD (mm)	Year	Method	N	Imaging Modality	Preprocessing
Post-operative glioblastoma multiforme segmentation with uncertainty estimation [68]	T1 enhancement (Whole Tumor) *	0.81	29.56	2022	3D nnUNet + manual uncertainty threshold	340 post-treatment patients (270 train, 70 test)	T1 post gadolinium contrast enhancement	Bias field correction + skull stripping
Segmentation of glioblastomas in early post-operative multi-modal MRI with deep neural networks [67]	Residual Tumor Volume *	0.5919	22.56 (95th HD)	2023	3D nnUNet	956 post-treatment patients (73 testing)	T1 + T1 post gadolinium contrast enhancement	Alignment
A Fully Automated Post-Surgical Brain Tumor Segmentation Model for Radiation Treatment Planning and Longitudinal Tracking [66]	Radiotherapy Targets (Gross Tumor Volume 1)	0.72	12.77	2023	3D UNet	255 patients (202 train, 23 validation, 30 test)	T1 post gadolinium contrast enhancement + T2 FLAIR	Skull stripping + alignment
Longitudinal Assessment of Posttreatment Diffuse Glioma Tissue Volumes with Three-dimensional Convolutional Neural Networks [10]	Whole Tumor Post-treatment	0.86	6.9 (95th HD)	2022	3D nnUNet	298 patients post-treatment (198 train, 100 test)	T1 + T1 post gadolinium contrast enhancement + T2 + T2 FLAIR	Skull stripping + alignment
Development and Practical Implementation of a Deep Learning–Based Pipeline for Automated Pre- and Postoperative Glioma Segmentation [77]	Whole Tumor Post-treatment	0.83	N/A	2022	Autoencoder regularization–cascaded anisotropic CNN	437 patients post-treatment (40 test, 397 training)	T1 + T1 post gadolinium contrast enhancement + T2 + T2-FLAIR	Skull stripping + alignment
A Deep Learning Approach for Automatic Segmentation during Daily MRI-Linac Radiotherapy of Glioblastoma [76]	Whole Tumor Post-treatment	0.67	N/A	2023	Mask R-CNN	36 patients (imaging pre- and 30 times during treatment totaling 930 images; 9-fold cross validation with 80:10:10 train:val:test)	Predominantly T2-weighting low field (0.35T) bSSFP	None
Towards Longitudinal Glioma Segmentation: Evaluating combined pre- and post-treatment MRI training data for automated tumor segmentation using nnU-Net [75]	Whole Tumor Post-treatment	0.8	N/A	2023	3D nnUNet	Pre-treatment training cases: (N = 502). Post-treatment training cases: (N = 588). Combined cases: (N = 1090). Test cases from pre-treatment: (N = 219); and post-treatment: (N = 254).	T1 post gadolinium contrast enhancement + T2 FLAIR	Alignment + denoting + N4 Bias correction + skull stripping
This manuscript	Whole Tumor Post-treatment	0.86	6.88 (95th HD)	2024	Transfer learning 3D VAE with spatial regularization	Pre-treatment training cases: (N = 208). Post-treatment training cases: (N = 197). Post-treatment test cases: (N = 24).	T2 FLAIR	None

* Only contrast-enhancing lesion, not to be compared with T2-lesion segmentation.

**Figure A1:**
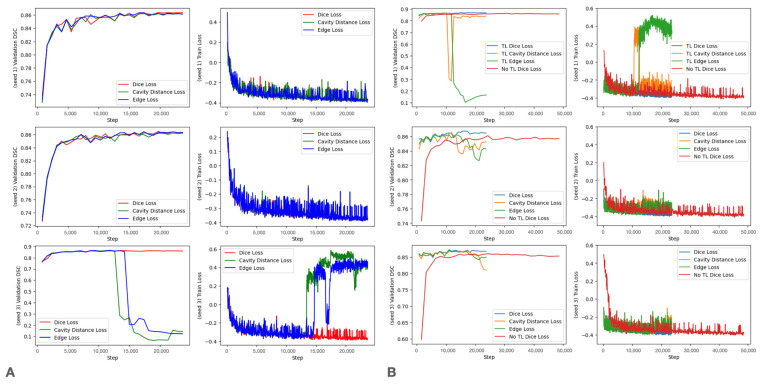
((**A**) **Left**) Validation Dice scores for all types of models across every random seed used for training and analysis of loss modification. ((**A**) **Right**) Training loss curves for all models across every random seed used for training and analysis of loss modification. ((**B**) **Left**) Validation Dice scores for all types of models across every random seed used for training and analysis of loss modification with transfer learning. ((**B**) **Right**) Training loss curves for all models across every random seed used for training and analysis of loss modification with transfer learning.

## Data Availability

The datasets presented in this article are not readily available because of patient privacy restrictions. Requests to access the datasets should be directed to the principal investigator of the study.

## Appendix A

### Scan Parameters

Images were acquired with a 3D T2-FLAIR sequence in axial or coronal planes and reformatted to axial orientation with a FOV of [256,256,210] and to the number of pixels of [256,256,140]. The original ranges of FOV and number of pixels are provided below in tabular format.

**Table A1:** Range of original T2 FLAIR acquisition resolutions.

	FOV	Number of Pixels
Min resolution	[205,205,257]	[256,256,208]
Max resolution	[179,179,249]	[256,256,214]

## Appendix B

### Generating Distance Maps for Weighting the Loss

The process for generating the edge distance map used a max normalization and inversion of the 3D Euclidean distance transform of the binary mask of the radiologist-annotated T2 lesion, followed by multiplication with the original mask and max normalization to generate a distance map. The resulting map weights pixels closest to the edges of the T2 lesion most highly and can be described as follows:

$$M_{{edt}_{i}}=[{\sum_{d=1}^{D=3}{\left({M_{lesion}x}_{d_{i}}-{M_{lesion}b}_{d_{i}}\right)}^{2}]}^{\frac{1}{2}}$$

$$M_{int}=|1-\frac{M_{edt}}{max\{M_{edt}\}}|\cdot M_{lesion}$$

$$M_{edge}=\frac{M_{int}}{max\{M_{int}\}}$$

where ${M_{lesion}x}_{d_{i}}$ are the i pixels of the binary mask; and ${M_{lesion}b}_{d_{i}}$ are the background pixels, with the smallest Euclidian distance in d = 3 dimensions; and $edt(M_{lesion})$ is the transform applied to the entire image. This preprocessing step was used to generate 158 edge maps from the post-treatment T2 lesion annotations that were used for training (data in validation and testing do not need the distance maps since the cross-entropy term is not used for early stopping criteria). An example edge map weighting scheme weighting is shown in Figure 1.

For the cavity distance penalizations, a similar preprocessing pipeline was used to generate the distance maps. To generate cavity lesions for 133 patients missing manual annotations, we first applied a ventricle blocking method by skull stripping, using the dilated T2 lesion to define a search area, and performing a series of thresholding and morphological operations. Next, the 3D Euclidean distance transform of an inverted manually annotated cavity mask was multiplied by the T2 lesion and max normalized. An inverted distance transform was then multiplied by the T2 lesion mask and max normalized before subtracting with the non-inverted transform map and re-normalizing. This process generated a distance map where pixels of the lesion closest and farthest away from the surgical cavity are weighted most highly, as shown in Figure 1, and described as follows:

$$M_{iedt}=edt(|1-M_{cavity}|)$$

$$M_{int1}=\frac{M_{iedt}\cdot M_{lesion}}{max\{M_{iedt}\cdot M_{lesion}\}}$$

$$M_{int2}=\frac{{|1-M}_{iedt}|\cdot M_{lesion}}{max\{{|1-M}_{iedt}|\cdot M_{lesion}\}}$$

$$M_{cavity\_distance}=\frac{\left|M_{int1}-M_{int2}\right|}{max\{{|M}_{int1}-M_{int2}|\}}$$
